# Emerging Glycation-Based Therapeutics—Glyoxalase 1 Inducers and Glyoxalase 1 Inhibitors

**DOI:** 10.3390/ijms23052453

**Published:** 2022-02-23

**Authors:** Naila Rabbani, Paul J. Thornalley

**Affiliations:** 1 Department of Basic Medical Science, College of Medicine, Qatar University Health, Qatar University, Doha P.O. Box 2713, Qatar; 2 Diabetes Research Center, Qatar Biomedical Research Institute, Hamad Bin Khalifa University, Qatar Foundation, Doha P.O. Box 34110, Qatar

**Keywords:** methylglyoxal, glyoxalase, dicarbonyl stress, diabetes, cancer chemotherapy, malaria, resveratrol, SARS-CoV-2

## Abstract

The abnormal accumulation of methylglyoxal (MG) leading to increased glycation of protein and DNA has emerged as an important metabolic stress, dicarbonyl stress, linked to aging, and disease. Increased MG glycation produces inactivation and misfolding of proteins, cell dysfunction, activation of the unfolded protein response, and related low-grade inflammation. Glycation of DNA and the spliceosome contribute to an antiproliferative and apoptotic response of high, cytotoxic levels of MG. Glyoxalase 1 (Glo1) of the glyoxalase system has a major role in the metabolism of MG. Small molecule inducers of Glo1, Glo1 inducers, have been developed to alleviate dicarbonyl stress as a prospective treatment for the prevention and early-stage reversal of type 2 diabetes and prevention of vascular complications of diabetes. The first clinical trial with the Glo1 inducer, *trans*-resveratrol and hesperetin combination (tRES-HESP)—a randomized, double-blind, placebo-controlled crossover phase 2A study for correction of insulin resistance in overweight and obese subjects, was completed successfully. tRES-HESP corrected insulin resistance, improved dysglycemia, and low-grade inflammation. Cell permeable Glo1 inhibitor prodrugs have been developed to induce severe dicarbonyl stress as a prospective treatment for cancer—particularly for high Glo1 expressing-related multidrug-resistant tumors. The prototype Glo1 inhibitor is prodrug S-p-bromobenzylglutathione cyclopentyl diester (BBGD). It has antitumor activity in vitro and in tumor-bearing mice in vivo. In the National Cancer Institute human tumor cell line screen, BBGD was most active against the glioblastoma SNB-19 cell line. Recently, potent antitumor activity was found in glioblastoma multiforme tumor-bearing mice. High Glo1 expression is a negative survival factor in chemotherapy of breast cancer where adjunct therapy with a Glo1 inhibitor may improve treatment outcomes. BBGD has not yet been evaluated clinically. Glycation by MG now appears to be a pathogenic process that may be pharmacologically manipulated for therapeutic outcomes of potentially important clinical impact.

## 1. Introduction–Dicarbonyl Stress in Health and Disease

**Figure 1:**
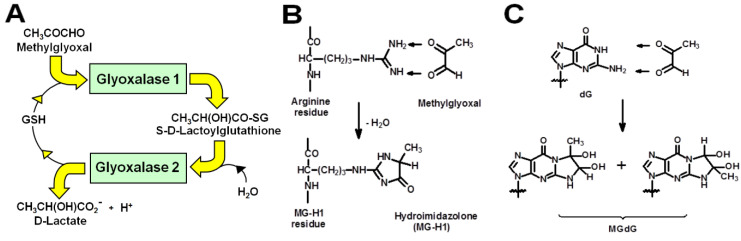
The glyoxalase system and protein and DNA glycation by methylglyoxal. (**A**) Metabolism of MG by the glyoxalase system. (**B**) Formation of hydroimidazolone MG-H1 from arginine residues. (**C**) Formation of imidazopurinone MGdG in DNA. Adduct residue is shown with guanyl base only.

Dicarbonyl stress is defined as the abnormal accumulation of dicarbonyl metabolites leading to increased protein and DNA modification contributing to cell and tissue dysfunction in aging and disease [1]. In physiological systems, a common reactive dicarbonyl metabolite is methylglyoxal (MG)—the major physiological substrate of the glyoxalase system (Figure 1A). In mammals, MG is mainly formed by the spontaneous degradation of triosephosphate intermediates of glycolysis, glyceraldehyde-3-phosphate, and dihydroxyacetonephosphate. MG is metabolized by glyoxalase 1 (Glo1) of the glyoxalase system in the cytosol of all cells. MG reacts spontaneously with reduced glutathione (GSH) to form a hemithioacetal and this is converted to S-D-lactoylglutathione by Glo1. S-D-Lactoylglutathione is hydrolyzed by glyoxalase 2 to D-lactate, reforming GSH consumed in the Glo1-catalysed step. MG that escapes metabolism may react non-enzymatically with proteins and DNA to form mainly arginine-derived hydroimidazolone, MG-H1, and deoxyguanosyl-derived imidazopurinone, MGdG, respectively (Figure 1B,C). MG-H1 and MGdG are major quantitative advanced glycation endproducts (AGEs) found in mammalian metabolism [2,3]. Metabolism of MG by Glo1 suppresses levels of MG-derived protein and DNA adducts to low, tolerable levels [1]. Human Glo1 is a highly efficient enzyme [4] and relatively high abundance protein—ca. 0.2 µg per mg total protein in human tissues [5], befitting its function as an important cytoprotective enzyme [1].

The formation of MG-H1 in proteins replaces a hydrophilic, positively charged arginine residue with an uncharged, hydrophobic MG-H1 residue and thereby produces misfolding and activation of the unfolded protein response (UPR) [6,7]. Activation of the UPR is a driver of insulin resistance and low-grade inflammation linked to the development of type 2 diabetes mellitus (T2DM) and the development of vascular complications of diabetes—diabetic nephropathy, retinopathy, peripheral neuropathy, and increased risk of cardiovascular disease. Insulin resistance is also linked to the development of non-alcoholic fatty liver disease (NAFLD) [8,9,10], chronic kidney disease [11,12], decline of respiratory function [13,14,15], cardiovascular disease, and aging [16,17,18]. Increased DNA damage by MG may activate the DNA damage response; and when this process exceeds the capacity for DNA repair, DNA replication stalls and replication catastrophe is activated and apoptosis or necrosis ensues—as reviewed [19]. Our recent research suggested that the spliceosome is also a target for protein modification by MG. Dysfunction of the spliceosome may also be an initiator of apoptosis in the cytotoxic antiproliferative activity of MG-linked dicarbonyl stress [20]. Dysfunctional spliceosomal activity—decreased and abnormal alternate splicing—is a feature of therapeutic and disease processes where dicarbonyl stress has been implicated [1]. For example, the mechanism of action of many anticancer drugs [21] and the pathogenesis of type 1 diabetes mellitus (T1DM), T2DM, and Alzheimer’s disease [22].

Functional genomics studies of Glo1 using cellular and animal models of disease with overexpression or silencing of Glo1 have indicated where small-molecule inducers of Glo1 expression or “Glo1 inducers” may have therapeutic benefit. This includes: prevention and early-stage reversal of T2DM through correction of insulin resistance and dysglycemia [23] and prevention and treatment of microvascular complications of diabetes [24,25,26]. Through correction of insulin resistance, there may be also therapeutic benefits expected in the prevention and treatment of NAFLD, chronic kidney disease, decline of respiratory health, cardiovascular disease, and aging—see above. In experimental studies of liver carcinogenesis, in the non-malignant state, Glo1 was identified as a tumor suppressor protein [27]—likely linked to some targets of MG glycation facilitating malignant transformation. Hence, the Glo1 inducer may find application in the chemoprevention of cancer. The rationale for intervention with Glo1 inducer is to correct a pathogenesis-associated decline in Glo1 expression and/or an increase in the formation of MG, leading to, typically, a 2–3-fold increase in plasma and tissue-specific increase in the concentration of MG [1,7,28]. Such moderate increase in MG is not associated with acute cytotoxicity, although it may induce low-level detachment of cells from the extracellular matrix and related detachment-stimulated apoptosis or anoikis [29] and may increase low level UPR-stimulated apoptosis [30]. Rather, it is associated with low-grade inflammation and cell tissue dysfunction driving the development of chronic disease or complications of metabolic, vascular, and other diseases—as reviewed [1].

The prospective treatment applications for Glo1 inhibitors are cancer chemotherapy, adjunct therapy of high Glo1-expressing, multidrug-resistant tumors, and malaria. The rationale for treatment is to induce an acute profound increase in MG to cytotoxic levels which has selective toxicity to rapidly proliferating tumors and malarial protozoa [31]. This was found in vitro for the cell-permeable Glo1 inhibitor prodrug, S-p-bromobenzylglutathione cyclopentyl diester (BBGD) [32,33]. From considerations of metabolic modeling of MG metabolism and potency of the active Glo1 inhibitor, S-p-bromobenzylglutathione (BBG), delivered into cells by BBGD, we predicted that to achieve potent inhibition of tumor cell growth, the cellular concentration of MG was increased over 12-fold for ca. 24 h of the cell growth cycle [19]. Selective increase in MG levels in response to inhibition of Glo1 is favored by a higher glycolytic rate and concomitant increased rate of MG formation—as indicated by the 10–30 fold increase in expression of hexokinase-2 (HK2) in some human tumors [34]. It is also favored by tumor hypoxia—producing a switch to anaerobic glycolysis and increase in MG formation; as was found in in vitro studies where the anti-proliferative cytotoxicity of BBGD was enhanced 60-fold in model hypoxia [20]. Together with the relatively low susceptibility of quiescent non-malignant cells to MG-induced cytotoxicity and the short treatment period required to achieve the antitumor response, this may account for the observed selective toxicity of cell-permeable Glo1 inhibitors to tumors—see below.

Herein, we review the development and early-stage pre-clinical and clinical evaluation of the prototype, optimized Glo1 inducer—*trans*-resveratrol and hesperetin in combination (tRES-HESP), and the optimized Glo1 inhibitor prodrug, BBGD, for their respective therapeutic applications.

## 2. Development and Applications of Glyoxalase 1 Inducers for Improved Metabolic and Vascular Health

As the increased formation of MG and development of dicarbonyl stress emerged as an important pathogenic mechanism linked to the development of vascular complications of diabetes [35,36], atherosclerosis, and coronary heart disease [37,38,39], strategies to decrease the cellular concentration of MG clinically were explored. Chemical scavenging agents such as aminoguanidine and phenacylthiazolium bromide were potent scavengers of MG but were found to be toxic and unstable, respectively [40,41]. A better and potentially effective approach is to increase the expression and activity of Glo1. Since Glo1 metabolizes MG catalytically at diffusion-limited rates, it is highly effective in countering MG-linked dicarbonyl stress [4]. Induction of Glo1 expression can be achieved by small-molecule activators of transcription factor nuclear factor-erythroid factor 2-related factor 2 (Nrf2). We identified and validated a functional antioxidant response element (ARE) in the GLO1 gene to which Nrf2 binds and increases Glo1 expression [42]. We screened small molecule activators of Nrf2 with hit criteria: increased transcriptional response at ≤5 μM without significant cytotoxicity to human aortal endothelial cells (HAECs) and BJ fibroblasts in primary culture. Dietary bioactive compound selection criteria were the ability to activate Nrf2 at concentrations achieved or likely achievable at tolerable doses clinically and, where available, experimental evidence of ability to decrease glycation and/or toxicity induced by MG. After screening a focused library of ca. 100 dietary bioactive compounds, the compound producing the highest maximal response E_max_ for GLO1-ARE transcriptional activity was *trans*-resveratrol (tRES) and the compound with the lowest median effective concentration, EC_50,_ was hesperetin (HESP). Combining these two compounds produced pharmacological synergism: with 5 µM HESP, the EC_50_ of tRES was 1.46 ± 0.10 µM [43]. This brought the Glo1 inducer activity of tRES to the clinically achievable concentration range [44]. Co-administration of tRES with HESP may also facilitate increased bioavailability of tRES by inhibition of intestinal glucuronosyltransferases by HESP [45].

tRES-HESP was evaluated for its effect on cell function in HAECs, BJ fibroblasts, and periodontal ligament fibroblasts (PDLFs) primary culture, and HMEC-1 endothelial and hepatocyte-like HepG2 cell lines in vitro [7,28,43]. In HAECs, tRES-HESP decreased basal expression of the receptor for advanced glycation endproducts (RAGE) and cell adhesion molecules, intracellular adhesion molecule-1 (ICAM-1), vascular cell adhesion molecule-1 (VCAM-1) and E-selectin, and secretion of the inflammatory mediator, interleukin-8 (IL8) [43]. In HAECs and the HMEC-1 endothelial cell line cultured in high glucose concentration, tRES-HESP prevented increased glucose metabolism, dicarbonyl stress, activation of the UPR, and increased secretion of IL8 [7]. In BJ fibroblasts, tRES-HESP increased basal cellular GSH and decreased basal expression of RAGE and matrix metalloproteinase-3 (MMP3)—also known as stromelysin-1 [43]. In hepatocyte-like HepG2 cells, tRES-HESP increased basal cellular GSH [43].

tRES-HESP was evaluated in an experimental model of impaired wound healing in diabetic *db/db* mice. Topical application of tRES-HESP on alternate days for 6 days accelerated wound healing, compared to vehicle control [46].

tRES-HESP was evaluated in a clinical study of healthy overweight and obese subjects. The study was a randomized, double-blind, placebo-controlled crossover study in 29 subjects—Health Aging Through Functional Food (HATFF or Hats-off) study [43]. Dosing was by oral capsule, once daily, containing 90 mg tRES-120 mg HESP or placebo. The treatment periods were 8 weeks with 6 weeks washout period between the crossover. There was high compliance to tRES-HESP treatment and urinary excretion of tRES and HESP metabolites was increased >2000-fold and >100 fold, respectively, compared to placebo. Dietary questionnaires taken during the study indicated food consumption was similar throughout the study and subjects were advised to maintain their usual dietary habits. Clinical safety indicators were normal at study entry and remained unchanged throughout the placebo and tRES-HESP treatment periods, indicating supplementation with tRES-HESP was well-tolerated. tRES-HESP treatment produced a 22% increase in Glo1 activity of peripheral blood mononuclear cells (PBMCs) of all subjects, compared to placebo in all subjects. The increase was 27% in highly overweight subjects—defined as subjects with BMI > 27.5 kg/m^2^, and 30% in obese subjects (with BMI ≥ 30 kg/m^2^). Most changes were found in highly overweight subjects and outcomes are presented for this subgroup. Concomitant with increased Glo1 activity, there was a 37% decrease in plasma MG post-supplementation with tRES-HESP but not with placebo. The flux of endogenously formed MG-H1 adducts was decreased by 14% with tRES-HESP treatment but not with placebo [43].

The physiological effects of tRES-HESP treatment were profound. Insulin resistance was assessed by the oral glucose insulin sensitivity (OGIS) index—deduced from a change in plasma concentrations of glucose and insulin during an oral glucose tolerance test (OGTT) and correlating strongly with the reference hyperinsulinemic-euglycemic clamp method [47]. OGIS index in overweight and obese subjects was corrected to levels typical of lean subjects with normal insulin sensitivity by treatment with tRES-HESP but not by placebo. The increase in OGIS of obese subjects was +58 mL min^−1^m^−2^ which is a change similar to that found with the pharmaceutical treatment of patients with T2DM (for example, 1.7 g metformin per day, ΔOGIS = +54 mL min^−1^m^−2^) [48] and extreme weight loss with gastric band surgery in morbid obesity (ΔOGIS = +62 mL min^−1^m^−2^) [49]. During treatment with tRES-HESP, change from baseline of PBMC Glo1 expression correlated negatively with change in area-under-the-curve plasma glucose during the OGTT (ΔAUCg). ΔAUCg correlated positively with PBMC expression of thioredoxin interacting protein (TXNIP); and change of PBMC expression of tumor necrosis factor-α (TNFα) correlated positively with change in fasting plasma glucose and negatively with a change in OGIS [50]. TXNIP is a mediator of insulin resistance in the liver, skeletal muscle, and adipose tissue and impaired pancreatic beta-cell insulin secretion [51,52,53] and TNFα decreases insulin receptor signaling in adipose tissue and skeletal muscle, particularly prior to the development of T2DM [54,55,56]. Correction of insulin resistance by tRES-HESP suggests this supplement may be appropriate for the prevention and early-stage reversal of T2DM [57,58]. Correction of insulin resistance is also a therapeutic target for the treatment of NAFLD [59] and may offer a further prophylactic application for tRES-HESP.

Treatment with tRES-HESP in the HATFF study also decreased low-grade inflammation, characterized by decreased expression of monocyte chemoattractant protein-1 (MCP-1), prostaglandin synthetase-2 or cyclo-oxygenase-2 (COX-2), interleukin-8 (IL8), and RAGE [43]. This anti-inflammatory effect has not been achieved in clinical studies with tRES alone [60,61]. If the anti-inflammatory effects of tRES-HESP on gene expression in PBMCs translate to tissues, there may be additional health benefits of tRES-HESP through a decrease in low-grade inflammation in NAFLD [8,9,10], chronic kidney disease [11,12], and decline of respiratory function [13,14,15], cardiovascular disease, and aging [16,17,18].

**Figure 2:**
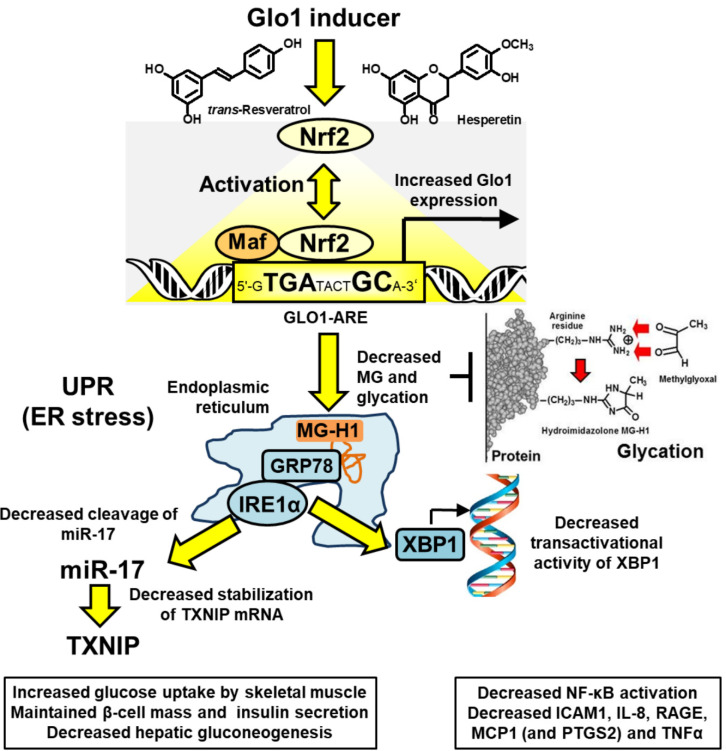
Proposed mechanism of action of Glo1 inducer, tRES-HESP, through suppression of the unfolded protein response. Key: yellow filled arrows—mechanism of health improvement by; red filled arrows—damaging processes suppressed. See text for details. Abbreviations: GRP78, 78 kDa glucose-regulated protein; IRE1α; inositol regulated enzyme-1α; Maf, basic region leucine zipper-type transcription factor; miR-17. microRNA-17; Nrf2, nuclear factor-erythroid factor 2-related factor 2; TXNIP, thioredoxin-interacting protein; XBP1, X-box binding protein-1. Modified and reproduced with permission from [50].

Upstream signaling of MCP-1, IL-8, RAGE, and COX-2 may be linked to activation of the UPR by increased MG-modified misfolded proteins [7,28] (Figure 2). In UPR activation, the exonuclease activity of inositol requiring enzyme-1α (IRE1α) cleaves microRNA, miR-17, and thereby stabilizes TXNIP mRNA to increase its expression and activity [62,63]. TXNIP decreases glucose uptake by skeletal muscle and pancreatic beta-cell mass and insulin secretion and increases hepatic gluconeogenesis [51,64,65]. Inflammatory signaling may be mediated through X box-binding protein 1 (XBP1), increasing histone H3 lysine 4 methyltransferase, SET7/9, expression of p65 of the NF-κB system and inflammatory mediators [66,67], including TNFα as a key contributor to insulin resistance in skeletal muscle [68,69]. Treatment with tRES-HESP alleviates these UPR-mediated responses [7,28]. Recent correlation analysis of data from the HATFF study indicated that tRES-HESP may be linked to improvements in dysglycemia, blood pressure, and dyslipidemia which may achieve significant change in larger clinical studies [50]. The pharmacological activity of tRES-HESP in pre-clinical disease models and clinical trials is summarized in Table 1.

The health benefits of tRES-HESP have not been found for tRES or HESP individually. For example, from a meta-analysis, it was concluded that tRES does not affect glycemic status in overweight and obese human subjects [70]. This is at odds with evidence from rodent models [71] and is likely due to interspecies differences in pharmacology, host interactions, and maximum tolerable dose. HESP absorbed from clinical dosing with hesperidin (hesperetin 7-rutinoside) neither improved plasma glucose nor insulin resistance [72]. The basis for synergism of the combination is likely an improvement in the bioavailability of tRES by inhibition of intestinal glucuronosyl transferases by HESP—as discussed previously—and pharmacological synergistic effects in the activation of Nrf2 [45]. Nrf2 is a constitutive translocational oscillator, with Nrf2 continually moving in and out of the cell nucleus [73]. The Nrf2 transcriptional response may be increased by increasing the frequency of the oscillations and slowing the inactivation of Nrf2 in the cell nucleus. HESP may activate Nrf2 by increasing the frequency of Nrf2 translocational oscillations by activation of protein kinase A, upstream of fyn kinase which has been proposed to phosphorylate Nrf2 for export from the cell nucleus [74,75]. tRES induces increased activity of NAD-dependent deacetylase, Sirtuin-1, and may thereby promote the removal of an inhibitory acetylation of Nrf2 to increase transcriptional activity [43,76]. The mechanism of activation of Nrf2 by tRES-HESP has been discussed elsewhere in this Special Series [77]. HESP is a partial agonist which is likely due to inhibitory nuclear acetylation of Nrf2 blocking a high E_max_. Combination with tRES and HESP provides faster nuclear translocation and decreased inactivation of Nrf2 [73,75,78]. The use of HESP rather than related dietary glycoside hesperidin found in citrus fruits [79] is likely also crucial as HESP has ca. 70-fold greater potency in Nrf2 activation and higher bioavailability than hesperidin [80].

There are likely off-target pharmacological effects of tRES-HESP that contribute to the therapeutic activity. For example, induction of expression of glucose-6-phosphate dehydrogenase (G6PD) and other ARE-linked genes [43]. An increase in expression of G6PD was implicated in a decrease in transcriptional activity of G6P/mlx/Mondo A complex regulating genes with a functional carbohydrate response element (ChRE)—including HK2, TXNIP, and other glycolytic and lipogenic genes. This corrected multiple pathways of metabolic dysfunction in model hyperglycemia in HAECs and PDLFs in vitro [7,28] and was implicated in the development of insulin resistance, vascular complications of diabetes, diabetic embryopathy, and ischemia-reperfusion injury in vivo [77,81]. Functional genomic studies with tissue-selective activation of Nrf2 by partial knockdown of Keap1 in the obesogenic HFD-fed mouse model of insulin resistance indicated that selective activation of Nrf2 in skeletal muscle and the liver corrected insulin resistance and dysglycemia, respectively [82]. This supports the findings of corrected insulin resistance and improved dysglycemia by treatment with tRES-HESP in the HATFF study [43]. Nevertheless, the effects of improved metabolic and vascular health in the HATFF study were achieved with the tRES-HESP dietary supplement by optimizing for induction of Glo1 expression.

tRES-HESP is a promising dietary supplement for further clinical evaluation—particularly for the prevention and treatment of T2DM and vascular complications of diabetes. It has the advantage of being highly tolerated without report of adverse effects which is desirable for intended chronic and prophylaxis treatment applications. Further evaluation is in progress.

## 3. Development and Application of Glyoxalase 1 Inhibitors for Cancer Chemotherapy

**Figure 3:**
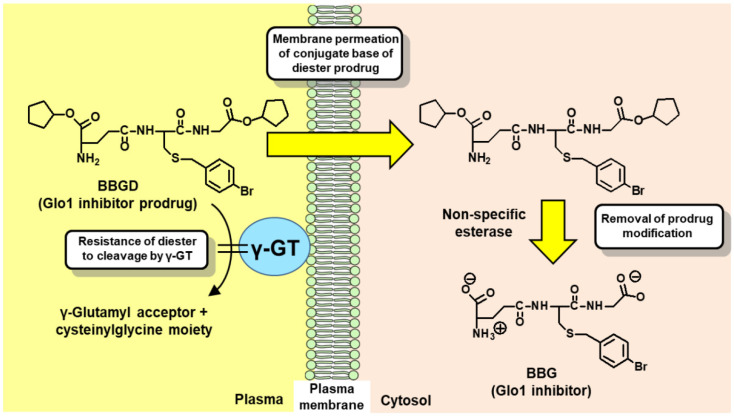
Delivery of glyoxalase 1 inhibitor, S-p-bromobenzylglutathione, into cells by diester modification. Abbreviations: BBG, S-p-bromobenzylglutathione; BBGD, S-p-bromobenzylglutathione cyclopentyl diester; and γ-GT, γ-glutamyl transferase.

The development of Glo1 inhibitors as therapeutic agents was first proposed by Vince and Wadd in 1969 [83]. Inhibition of Glo1 leads to the cellular accumulation of glyoxal and MG [32,84]. In 1967, Apple and Greenberg published studies on the antitumor activity of MG in tumor-bearing mice [13]. The antitumor activity of MG was relatively weak. This was considered due to the metabolism of MG by the glyoxalase system. Vince and Wadd reasoned that inhibition of Glo1 would likely increase endogenous MG to cytotoxic levels and proposed Glo1 inhibitors as a potential new class of anticancer drugs [83]. The initial inhibitors developed were competitive, substrate analog inhibitors of Glo1: S-alkylglutathione and S-benzylglutathione derivatives [85,86]. One of the most potent inhibitors was BBG [85] with an inhibitor constant K_i_ of 160 nM for human Glo1 [87]. However, potent antitumor activity was not achieved by treatment with BBG. Our team realized that this was likely due to poor membrane permeability of BBG and susceptibility of it to cleavage and degradation by cell surface γ-glutamyl transferase (γ-GT). Both of these characteristics could be countered by the diesterification of BBG on the carboxylate groups of the γ-glutamyl and glycyl residues. BBG diester has only one ionized group—the γ-glutamyl amino group, and thereby a unionized conjugate base in solution which is membrane permeable. Once the BBG diester is inside cells, the ester groups are hydrolyzed by cellular non-specific esterases to form the active inhibitor. The diester is also resistant to cleavage by γ-GT. This prodrug modification was key to achieving antitumor activity of specific substrate analog Glo1 inhibitors [32,88] (Figure 3).

We evaluated a range of BBG diester derivatives and the cyclopentyl diester, BBGD, gave the best balance of the ester groups being sufficiently resistant to cleavage by plasma non-specific esterases for delivery into cells whilst still being hydrolyzed to BBG when inside cells [89]. The median growth inhibitory concentration GC_50_ of BBDG with human leukemia 60 cells in vitro was 4.23 ± 0.01 µM [32] and in the National Cancer Institute (Bethesda, MD, USA) panel of human tumor cell lines, BBGD was active against leukemia, lung, colon, central nervous system, melanoma, ovarian, renal, prostate cancer, and breast cancer cell lines [90]. It was most potent for glioblastoma cell line, SNB-19 (Table 2). Creighton and co-workers developed more potent Glo1 inhibitors, including bidentate-spacer-linked substrate analog inhibitors that bound across the two active sites of dimeric human Glo1 [91]. They also recognized that for evaluation in tumor-bearing mice, esterase-deficient mice were required as conventional wild-type mouse strains have markedly higher plasma esterase activity than human subjects. The use of conventional wild-type strains of laboratory mice would underestimate the clinical potency of the prospective new drugs by rapid de-esterification in plasma [92]. Even so, BBGD had antitumor activity in tumor-bearing mice and was particularly effective against tumors with high Glo1 expression and resistance to established anticancer drugs [32,93]. Similar studies were performed with Glo1 competitive inhibitor S-(N-p-chlorophenyl-N-hydroxycarbamoyl)glutathione (CHG), K_i_ for human Glo1 of 0.046 µM [94], administered as prodrug CHG ethyl diester and cyclopentyl diester [95]. From efficacy studies, dosing schedules have been achieved that gave similar potency to current clinical antitumor agents [32,93,95]. Recent studies with xenograft implants of glioblastoma multiforme cells in mice found a profound decrease in tumor volume with two doses of 50 mg/kg BBGD [96] (Table 3). This may indicate that this tumor type is a candidate for the initial clinical evaluation of BBGD.

In 2000, working on a transcriptome-wide study of gene expression in cell lines sensitive and resistant to anticancer drugs, Takashi Tsuruo and colleagues—who discovered the role of P-glycoprotein in multidrug resistance (MDR) in cancer chemotherapy [101], found a further important factor in cancer chemotherapy MDR was increased Glo1 expression [102]. Subsequent studies by this team showed that co-treatment with BBGD re-established sensitivity to antitumor drugs in Glo1-linked MDR in human tumor cell lines. They also showed BBGD has antitumor activity in tumor-bearing mice [102].

In 2010, our team, working with Mike Stratton and colleagues of the UK Cancer Genome, reported on copy number increase of GLO1 in human tumors—a mechanism of increased Glo1 expression in some tumors [99]. Human GLO1 is located at locus 6p21.2 with low-level duplication in the healthy population of 2% prevalence [103]. The DNA segment copied in tumor GLO1 copy number increase was larger than in gene duplication in the healthy population [104]. In clinical tumors, GLO1 copy number was functional—associated with increased Glo1 mRNA and protein. In our study of 225 human tumors of different types, the highest prevalence of GLO1 copy number increase was in breast cancer (22%), sarcomas (17%), and non-small cell lung cancer (NSCLC) (11%) [99]. Further studies have found GLO1 amplification at very high prevalence in breast cancers that do not express estrogen receptor, progesterone receptor and HER-2 genes, triple-negative breast cancer (TNBC) [105], advanced malignant melanoma [106], and neuroendocrine tumors where increased GLO1 copy number was associated with poor patient survival [107]. The mechanism of GLO1 amplification in cancer is unknown but a suggestion came from a study of GLO1 duplication in mouse embryonic stem cells [108]. A low-level copy number increase in GLO1 was found in hypoxia, suggesting a role for hypoxia-activated histone demethylase, lysine demethylase 4A (KDM4A). The Glo1 gene is present in a large transcriptional domain, in the flanking region of BTBD9 in which copy number increases are enriched. Black et al. reported a genetic domain-specific increase in gene copy number in primary human T-lymphocytes and tumors—including genes linked to MDR—driven by KDM4A [109,110]. Increased histone demethylation is hypothesized to create more open chromatin which promotes inappropriate recruitment of mini-chromosome maintenance proteins and DNA polymerases and thus promote re-replication for copy number gain [110]. Tumor hypoxia may activate KDM4A demethylation to drive increased GLO1 copy number. Human tumor cell lines with increased GLO1 copy number and Glo1 expression were generally more susceptible to cytotoxic effects of silencing of Glo1 with siRNA and treatment with BBGD [99].

In recent studies, we identified the likely cause of Glo1-linked MDR in cancer chemotherapy. Multiple classes of clinical anticancer drugs—alkylating agents, topoisomerase inhibitors, anti-tubulins, and anti-metabolites—increased the cellular concentration of MG to cytotoxic levels by off-target effects on glycolysis, increasing the flux of MG formation. The effects of anticancer drugs on glycolysis include: doxorubicin increases expression of glucose transporter GLUT1 and HK2 [111]; paclitaxel stabilizes microtubules, decreasing free tubulin concentration which increases mitochondrial voltage-dependent anion channel activity and thereby in situ activity of hexokinase-1 and HK2 [112]; methotrexate activates AMP-activated protein kinase and thereby stimulates glycolysis by activating phosphorylation of 6-phosphofructo-2-kinase/fructose-2,6-bisphosphatase [113]; and alkylating agents such as mechlorethamine and topoisomerase inhibitors lead to decreased cellular NAD^+^ in early-stage treatment, decreasing glycolysis at the glyceraldehyde-3-phosphate dehydrogenase-catalyzed step and consequently increasing cellular glyceraldehyde-3-phosphate and formation of MG [114,115,116]. Overexpression of Glo1 countered this to decreased cytotoxic antiproliferative activity of antitumor drugs. The antiproliferative activity of BBGD was increased ca. 60-fold in model hypoxia by increasing anaerobic glycolysis and the flux of MG formation and decreasing expression of Glo1 through activation of hypoxia-inducible factor-1α. We explored how an increased MG concentration induced tumor cell death and found it activated the intrinsic pathway of apoptosis, with a decrease in mitochondrial and spliceosomal proteins. Spliceosomal proteins were targets of MG modification. Spliceosomal gene expression correlated positively with Glo1 in human tumor cell lines and tumors. High expression of Glo1 may contribute to MDR by shielding the spliceosome from MG modification and decreasing survival in chemotherapy. Therefore, MG-mediated cytotoxicity appears to contribute to the cancer chemotherapeutic response by targeting the spliceosome [20].

Following these recent findings, it may be timely to develop further Glo1 inhibitors to specifically increase MG concentration for antitumor activity—particularly for tumors such as TNBC where there is a high prevalence of increased Glo1 copy number, high Glo1 expression, and current chemotherapy is ineffective. In clinical chemotherapy of breast cancer, increased expression of Glo1 was associated with decreased patient survival, with hazard ratio HR = 1.82 where the upper quartile survival of patients was decreased 64% with high Glo1 expression; upper quartile overall survival decreased from ca. 15 to 5 years [20]. Primary or adjunct chemotherapy with Glo1 inhibitor to counter Glo1-mediated MDR may improve treatment outcomes [20] and high Glo1 expression may be a biomarker of likely tumor susceptibility to Glo1 inhibitor therapy [99].

## 4. Other Potential Therapeutic Applications and Experimental Applications of Cell Permeable Glyoxalase 1 Inhibitors

Other potential therapeutic applications of cell-permeable Glo1 inhibitors involve pathogenic microbial infections where the microbial life cycle has a stage with high anaerobic glycolytic activity and high Glo1 expression. An example is an infection with malaria protozoa.

Glo1 inhibitor was evaluated for anti-malarial activity against *Plasmodium falciparum* malarial parasite-infected red blood cells (RBCs) in vitro. The RBC stage of the malarial parasite life cycle has only functional anaerobic glycolysis [117] and the rate of formation of MG in human RBCs increases by ca. 10–20-fold upon infection with *P. falciparum* [118]. S-p-Bromobenzylglutathione diethyl ester had potent antimalarial activity against *P. falciparum* in RBC cultures, inhibiting parasite nucleotide and protein synthesis with median inhibitory concentrations (IC_50_) of 4.8 µM and 5.2 µM, respectively. With 6 µM Glo1 inhibitor, parasite nucleotide synthesis was totally inhibited after treatment for 6 h [33]. Later studies with p-bromobenzyl-hydroxamic acid substrate analog inhibitors of Glo1 gave IC_50_ values of 30–35 µM but had increased cytotoxicity compared to S-bromobenzylglutathione diester [119].

We recently proposed an evaluation of BBGD for antiviral activity against SARS-CoV-2 [120]. Antiviral activity of supraphysiological concentrations of MG was reported historically [121], with inhibition of the cytopathic effect of influenza B strains by MG where the most sensitive strain gave a median inhibitory concentration of 23 ± 7 µM MG [122]. We explored the SARS-CoV-2 proteome for the enrichment of arginine residues—the major target of MG modification—in functional domains. We found a 4.9-fold enrichment of arginine residues in functional domains, suggesting the SARS-CoV-2 proteome may be susceptible to functional inactivation by MG and Glo1 inhibitors may have SARS-CoV-2 antiviral activity. We found both the spike protein and nucleoprotein had arginine residues potentially reactive towards MG [120]. Studies of antiviral activity of BBGD against SARS-CoV-2 are ongoing.

BBGD has become a leading research tool to explore the effect of pharmacologically induced dicarbonyl stress in experimental studies. It is commercially available and is now widely used. In addition to the applications to the chemotherapy of cancer and malaria described above, it has been used in studies exploring the effect of dicarbonyl stress in atherosclerosis [123], diabetes and vascular complications of diabetes [26,84,124], and cell models of pathogenesis in NAFLD [125], anxiety-linked behavior [126], osteoporosis [127] and age-related decline in heart function [128].

## 5. Concluding Remarks, Future Perspective and Limitations

There are now pharmacological agents to investigate experimentally and clinically the therapeutic outcomes of increasing expression of Glo1 with Glo1 inducer, tRES-HESP. The major therapeutic applications for Glo1 inducers are prevention, reversal, and treatment of T2DM and prevention and treatment of vascular complications of diabetes.

For the future perspective, Glo1 inducers now merit further clinical evaluation where correction of insulin resistance and a decrease in dicarbonyl stress are likely key to achieving therapeutic outcomes—particularly studies to evaluate tRES-HESP for early-stage reversal of T2DM. Reversal or remission of T2DM is now a key part of diabetes prevention programs [129]. Although progress has been made in the reversal of T2DM with low and very low calorie diets, there are some patients with T2DM who do not respond to these interventions and there is also uncertainty over compliance to dietary restriction and maintenance of T2DM reversal in a primary care setting [130]. Correction of insulin resistance is considered key to the prevention and reversal of T2DM [131] and tRES-HESP achieved this in the HATFF study without caloric or other dietary restriction [43]. tRES-HESP may offer an additional treatment option to prevent and reverse T2DM. This is particularly important in countering the current global record high prevalence of T2DM [132]. Vascular complications of diabetes are also a therapeutic target for the application of Glo1 inducers. There is currently no effective treatment for diabetic neuropathy which affects approximately 50% of patients with diabetes [133]. This is a priority disease target for the evaluation of tRES-HESP in pre-clinical and clinical studies.

A limitation of Glo1 inducer tRES-HESP is that there are multiple pharmacological benefits: on-target response—increasing expression of Glo1; and off-target responses—increasing expression of G6PD, decreasing expression of HK2, and others. It is a limitation in the sense that it is not clear which of these effects is the most important to optimize for health benefits—although by optimizing induction of expression of Glo1, we arrived at the combination of tRES-HESP—a supplement that had profound health benefit in a clinical trial [43]. Further investigation is also required to fully understand the mechanism of action by which tRES and HESP activate Nrf2. Plausible receptors have been proposed [45,77].

The cell-permeable Glo1 inhibitor, BBGD, is available to evaluate the therapeutic outcomes of inhibiting Glo1. The major therapeutic application for Glo1 inhibitors is cancer chemotherapy—particularly tumors with high Glo1 expression and adjunct therapy with cancer chemotherapy to counter Glo1-linked MDR, where clinical evaluation against glioblastoma multiforme and breast cancer appears to be promising treatment targeting tumor types.

For the future perspective, a key advance will be the first clinical trial of a Glo1 inhibitor in cancer chemotherapy. Recent research showing the potent activity of BBGD against glioblastoma tumor-bearing mice [96] suggests this type of tumor may be the optimum target for BBGD clinically. Glioblastoma is the most common brain tumor, representing ca. 48% of all primary tumors of the central nervous system, and current treatment is ineffective with a 5-year survival of only 5.8% [134]. A clinical trial with a Glo1 inhibitor could provide a potential breakthrough for chemotherapy of glioblastoma.

The limitations of Glo1 inhibitor therapeutics for cancer chemotherapy are the requirement of high glycolytic rate and high Glo1 expression of the tumor target. However, many tumors meet these criteria [19,20,34]. Therefore, there is potential therapeutic benefit from further development and clinical evaluation of Glo1 inhibitors for cancer chemotherapy. There are expected to be some adverse effects of BBGD—such as hematologic and other toxicities [135]—but initial studies with proliferating lymphocytes in vitro and tumor-bearing mice suggested adverse effects are limited [32].

Both Glo1 inducers and Glo1 inhibitors now offer the opportunity for important therapeutic advances in areas of unmet clinical need and major therapeutic impact.

## Figures and Tables

**Table 1:** Pharmacological activity of glyoxalase 1 inducer, *trans*-resveratrol and hesperetin, in pre-clinical disease models and clinical trial.

Application	Evaluation Model	Main Outcome	Reference
GLO1-ARE transcriptional activity	Stable transfectant luciferase reporter cell lines with GLO1-ARE or functionally inactive mutant as negative control	tRES: EC_50_ = 2.52 ± 0.19 µM and E_max_ = 100 ± 2% HESP: EC_50_ = 0.59 ± 0.01 µM and E_max_ = 24.4 ± 0.1% With 5 µM HESP, for tRES: EC_50_ = 1.46 ± 0.10 µM and E_max_ = 95.0 ± 0.1%	[43]
Cell vitality markers	Human aortal endothelial cells (HAECs) in primary culture	Decreased glucose metabolism, RAGE, ICAM-1, VCAM-1, E-selectin, and IL8 secretion.	[7,43]
	Human fibroblasts in primary culture	Increased cellular GSH and decreased RAGE and MMP3	[43]
	Human hepatocyte-like HepG2 cells in vitro	Increased cellular GSH	[43]
Endothelial cell dysfunction in diabetes	Human aortal endothelial cells (HAECs) in primary culture	In high glucose concentration, tRES-HESP (10 μM) corrected HK2-linked glycolytic overload, metabolic dysfunction and IL8 secretion	[7]
Fibroblast dysfunction in diabetes	Human periodontal ligament	In high glucose concentration, tRES-HESP (10 μM) corrected HK2-linked glycolytic overload, metabolic dysfunction and adhesion to extracellular matrix	[28]
Wound healing in diabetes	Dermal wound healing by topical application of tRES-HESP on alternate days for 6 days in db/db mice	tRES-HESP (5 μM) accelerated wound healing, compared to vehicle control	[46]
Metabolic and vascular health	Randomized, double-blind placebo-controlled crossover study in overweight and obese subjects (*n* = 29). Treatment was for 8 weeks, once-daily by oral capsule containing 90 mg tRES and 120 mg HESP or placebo, with 6 weeks washout (HATFF study; NCT02095873)	Effect of tRES-HESP: target pharmacology—PBMC activity of Glo1 (+27%), and plasma MG concentration (−37%); clinical endpoint-related variables—FPG (−5%), AUCg (−8%) and OGIS (+54 mL min^−1^m^−2^); and other—decreased expression of MCP-1, IL-8, COX-2 and RAGE in PBMCs. Urinary excretion of tRES and HESP metabolites increased >2000-fold and >100 fold, respectively. Placebo had no effect.	[43]

**Table 2:** Anti-cancer activity of cell-permeable Glo1 inhibitor, S-p-bromobenzylglutathione cyclopentyl diester, with human tumor cells lines in vitro.

Cell Lines	Main Outcome	Reference
Human leukemia 60 (HL60)	GC_50_ = 4.2 µM; cf. GC_50_ = 8.3 µM for ethyl diester and GC_50_ of 4.2–29.2 µM for series of BBG alkyl and cycloalkyl diesters with BBGD fund to be most potent	[32,88,89]
National Cancer Institute anticancer screen of 60 human tumor cell lines	GC_50_ = 5–20 µM for leukemia, NSCLC, colon, CNS, melanoma, ovarian, renal, prostate and breast cancer cell lines—most potent for glioblastoma SNB-19 (data for ethyl diester)	[90]
A549, DMS114, DMS273, NCI-H23, NCI-H226, NCI-H460 and NCI-H522 cell lines	GC_50_ range 4.4–29.7 µM	[93]
Sixteen gastric tumor cell lines	GC_50_ range 3–10 µM	[97]
Hepatocellular carcinoma HUH7	Inhibition of cell growth at 1–10 µM	[98]
Glioblastoma multiforme T98 and U87	GC_50_: T98, 100.6 µM; and U87, 9.9 µM	[96]
Osteosarcoma MG63; lung adenocarcinoma A549, NCI-H522 and NCI-H460; pancreatic carcinoma YAPC; squamous cell carcinoma LB771; and brain astrocytoma CCF-STTG-1	GC_50_: MG63, 3.8 µM; A549 23.5 µM; NCI-H522, 7 µM; NCI-H460, 19.8 µM; YAPC, 10 µM; LB771, 9.5 µM; and CCF-STTG-1, 1 µM	[99]
FaDu hypopharyngeal carcinoma and CAL27 oral adenosquamous carcinoma	GC_50_ ca. 3 µM	[100]

**Table 3:** Anti-cancer activity of cell permeable Glo1 inhibitor, S-p-bromobenzylglutathione cyclopentyl diester, in tumor-bearing mice in vivo.

Tumor Bearing Mouse Model	Main Outcome	Reference
Xenografts of lung cancer DMS114 and prostate cancer DU-145 s.c. in nude mice. Dosing: BBGD (100 mg/kg/day) i.p. from day 0 to 8.	40–50% inhibition of tumor growth.	[93]
Adenocarcinoma 15A cells s.c. in mice. Dosing: BBGD (50–200 mg/kg) administered i.p. at day 4 post-implant. Tumor mass recorded at day 7 post-treatment	50–200 mg/kg BBGD decreased tumor volume by 30–42%	[32]
Glioblastoma multiforme (GMB) orthotopic xenograft mouse model U87 glioma cells expressing ZsGreen1-firefly luciferase brain tumor xenograft implants in non-obese diabetic/severe combined immunodeficiency mice. Dosing: BBGD (50 mg/kg), i.p. on days 13 and 15 post-implant	Profound decrease in tumor volume at day 17 post-implant. Total tumor volume: vehicle, 4.6 × 10^11^ µm^3^; and BBDG-treated, 1.33 × 10^2^ µm^3^ (>>99.9% decrease; *p* < 0.0001)	[96]
*cf. Treatment with S-(N-p-chlorophenyl-N-hydroxycarbamoyl)glutathione ethyl diester (CHGD)*		
C57BL/6 (Es-1^e^) esterase deficient mice with murine B16 melanoma, human prostate PC3 and human colon HT-29 adenocarcinoma. Dosing: i.v. bolus of 80 or 120 mg/kg CHGD, 5 days for 2 weeks or continuous infusion.	Potency was achieved similar to clinical antitumor drugs: Doxorubicin for B16, cisplatin for PC3 and vincristine for HT-29.	[95]

## Data Availability

Data sharing is not applicable to this article.

## Abbreviations

AGEs, advanced glycation endproducts; ARE, antioxidant response element; BBG, S-p-bromobenzylglutathione; BBGD, S-p-bromobenzylglutathione cyclopentyl diester; BTBD9, BTB domain containing 9; CHG, S-(N-p-chlorophenyl-N-hydroxycarbamoyl)glutathione; CHGD, S-(N-p-chlorophenyl-N-hydroxycarbamoyl) glutathione ethyl diester; ChRE, carbohydrate response element; COX-2, cyclo-oxygenase-2; G6PD, glucose-6-phosphate dehydrogenase; Glo1, glyoxalase 1; GSH, glutathione; γ-GT, γ-glutamyl transferase; HAEC, human aortal endothelial cell; HER-2, human epidermal growth factor receptor 2; HESP, hesperetin; HK2, hexokinase-2; ICAM-1, intracellular adhesion molecule-1; IL8, interleukin-8; IRE1α, inositol requiring enzyme-1α; demethylase, KDM4A, lysine demethylase 4A; MCP-1, monocyte chemoattractant protein-1; MDR, multidrug resistance; MG, methylglyoxal; MG-H1, arginine-derived hydroimidazolone (Nδ-(5-hydro-5-methyl-4-imidazolon-2-yl)-ornithine); MGdG, deoxyguanosyl-derived imidazopurinone (3-(2′-deoxyribosyl)-6,7-dihydro-6,7-dihydroxy-6/7-methylimidazo-[2,3-b]purine-9(8)one isomers and related structural isomers); MMP3, matrix metalloproteinase-3; NAFLD, non-alcoholic fatty liver disease; Nrf2, nuclear factor-erythroid factor 2-related factor 2; NSCLC, non-small cell lung cancer; OGIS, oral glucose insulin sensitivity; OGTT, oral glucose tolerance test; PBMC, peripheral blood mononuclear cell; PDLF, periodontal ligament fibroblasts; RAGE, receptor for advanced glycation endproducts; RBC, red blood cell; T1DM, type 1 diabetes mellitus; T2DM, type 2 diabetes mellitus; tRES, trans-resveratrol; tRES-HESP, trans-resveratrol and hesperetin combination; TNBC, triple negative breast cancer; TNFα, tumor necrosis factor-α; TXNIP, Thioredoxin-interacting protein; UPR, unfolded proteins response; VCAM-1, vascular cell adhesion molecule-1; XBP1, X box-binding protein 1.
